# Optimizing radiotherapy protocols using computer automata to model tumour cell death as a function of oxygen diffusion processes

**DOI:** 10.1038/s41598-017-01757-6

**Published:** 2017-05-23

**Authors:** Perrine Paul-Gilloteaux, Vincent Potiron, Grégory Delpon, Stéphane Supiot, Sophie Chiavassa, François Paris, Sylvain V. Costes

**Affiliations:** 1 Structure Fédérative de Recherche François Bonamy, Micropicell, CNRS, INSERM, Université de Nantes, Nantes, France; 2 CRCINA, INSERM, CNRS, Université de Nantes, Nantes, France; 3 Institut de Cancérologie de l’Ouest, Saint-Herblain, F-44800 France; 4 Biosciences, Lawrence Berkeley National Laboratory, MS:977, Berkeley, 94720 CA USA; 5 NASA Ames Research Center, Moffett Blvd, Mountain View, 94035 CA USA

## Abstract

The concept of hypofractionation is gaining momentum in radiation oncology centres, enabled by recent advances in radiotherapy apparatus. The gain of efficacy of this innovative treatment must be defined. We present a computer model based on translational murine data for *in silico* testing and optimization of various radiotherapy protocols with respect to tumour resistance and the microenvironment heterogeneity. This model combines automata approaches with image processing algorithms to simulate the cellular response of tumours exposed to ionizing radiation, modelling the alteration of oxygen permeabilization in blood vessels against repeated doses, and introducing mitotic catastrophe (as opposed to arbitrary delayed cell-death) as a means of modelling radiation-induced cell death. Published data describing cell death *in vitro* as well as tumour oxygenation *in vivo* are used to inform parameters. Our model is validated by comparing simulations to *in vivo* data obtained from the radiation treatment of mice transplanted with human prostate tumours. We then predict the efficacy of untested hypofractionation protocols, hypothesizing that tumour control can be optimized by adjusting daily radiation dosage as a function of the degree of hypoxia in the tumour environment. Further biological refinement of this tool will permit the rapid development of more sophisticated strategies for radiotherapy.

## Introduction

The ability of ionizing radiation (IR) to control tumour growth through the induction of DNA damage is the primary basis of radiation therapy strategies. Unrepaired DNA damage can lead to mitotic cell death or proliferation blockade of malignant cells^1^. To limit radiation toxicity, conventional radiation treatment delivers a daily 2 Gy fraction, which provides enough time for the healthy tissue to repair damage while the tumour, which receives a higher dose, is unable to complete repair^1^. Though successful for most individuals, conventional radiotherapy may be limited in some hypersensitive patients, who suffer from toxicity in surrounding normal tissues, or in some patients whose tumours develop radiation resistance^1^. Some of these issues have been somewhat ameliorated in the past ten years, with technical breakthroughs in tumour imaging and medical physics leading to better targeting of the tumour using the latest generation of image-guided-, intensity-modulated radiotherapy or proton-therapy^2, 3^. Better tumour targeting and reduced normal tissue irradiation open the door to more radical protocols where the dose per fraction may be significantly increased, and all fractions given over a shorter period, typically one or two weeks. In addition, new protocols may benefit from recent discoveries in molecular and cellular radiobiology which are often neglected in conventional radiotherapy. Common radio-oncology studies have already shed light on important radiation-induced processes such as mitotic death and cell cycle inhibition^1^. In addition, cancer cell signalling pathways, micro-environmental changes modifying endothelial cell behaviour, tissue oxygenation, and immune responses are all important factors that should be considered in the tumour response during radiotherapy^4^.

However, moving to new radiation protocols is complicated, as there is no optimal statistical characterization of the outcome and physicians will be reluctant to risk to have worse outcomes than those from conventional approaches. How can we quickly build confidence in new approaches, optimize selection of protocols, and compete with 70 years of knowledge in conventional therapy? Until now, medical strategies to optimize radiotherapy have been essentially based on clinical trials using assay/error escalation of doses and computational dosimetry searching for an optimized balance between high tumour control probability (TCP) and low normal tissue complication probability (NTCP)^1^. However, clinical trials are costly and increasingly more difficult to launch. Furthermore, the research scope in each trial remains limited, and cannot cover every new potential optimization or tumour type.

This article describes a method to bridge the gap between basic knowledge in radiation biology and the establishment of optimized radiotherapy protocols by introducing cellular automata models that model *in silico* the response of tumour cells and their micro-environment to ionizing radiation. As a first step for proof-of-principle, we develop a model which models a well described *in-vivo* situation in mouse, where orthotopic transplants of human prostate tumours are treated with radiation^5^.

With increasing computer power, non-deterministic stochastic models of cell behaviour are now able to model in a reasonable amount of time the tumour response to various insults, allowing computational biologists to conduct *in silico* clinical trials. In this model, individual tumour cells are independent automata evolving in an oxygenated environment perfused by blood vessels. The integration of our biological knowledge into this model allowed us to better apprehend the complex spatiotemporal relationship between oxygen and tumour cell death as the blood vessels accumulate damage after each fraction delivered to the tumour. After validating the model parameters for the simple vascular microenvironment of orthotopic tumours in nude mice, we predict TCP curves for such *in vivo* conditions under various hypotheses related to changes in blood vessel properties following exposure to fractionated doses of radiation. Our predictions and biological assumptions illustrate clearly the importance of hypoxia and blood vessel damage during radiotherapy. These results give a glimpse of what is currently lacking from empirical and one-size-fits-all radiotherapy protocols. Our model opens the prospect of more elaborate models which could eventually predict the response of individual human tumours in real clinical situations.

## Methods

### Orthotopic tumour data

All biological data were extracted from previously published manuscripts, especially Potiron *et al*.^5^. Briefly, PC3 tumours were transplanted in mice as described. Tumours were exposed to 2 Gy daily for 2 weeks to measure the biological impact of a conventional radiotherapy protocol using small animal radiotherapy and macroscopic and microscopic imaging tools (Faxitron CP-160, Faxitron X-Ray Corp, Wheeling, IL, USA). All experiments were performed in accordance with relevant guidelines and regulations as described in a previously published article^5^.

### Cellular automaton for tumour growth model

In order to study the response of tumours to radiotherapy and the impact of the tumour microenvironment (vascularization and oxygen levels) on the radio-sensitivity of tumours cells, a 2D and a 3D cellular automaton model was implemented. We describe the 2D model here, and demonstrate its equivalence to a 3D model using an extrapolation factor, while considerably economizing computation time.

**Figure 1:**
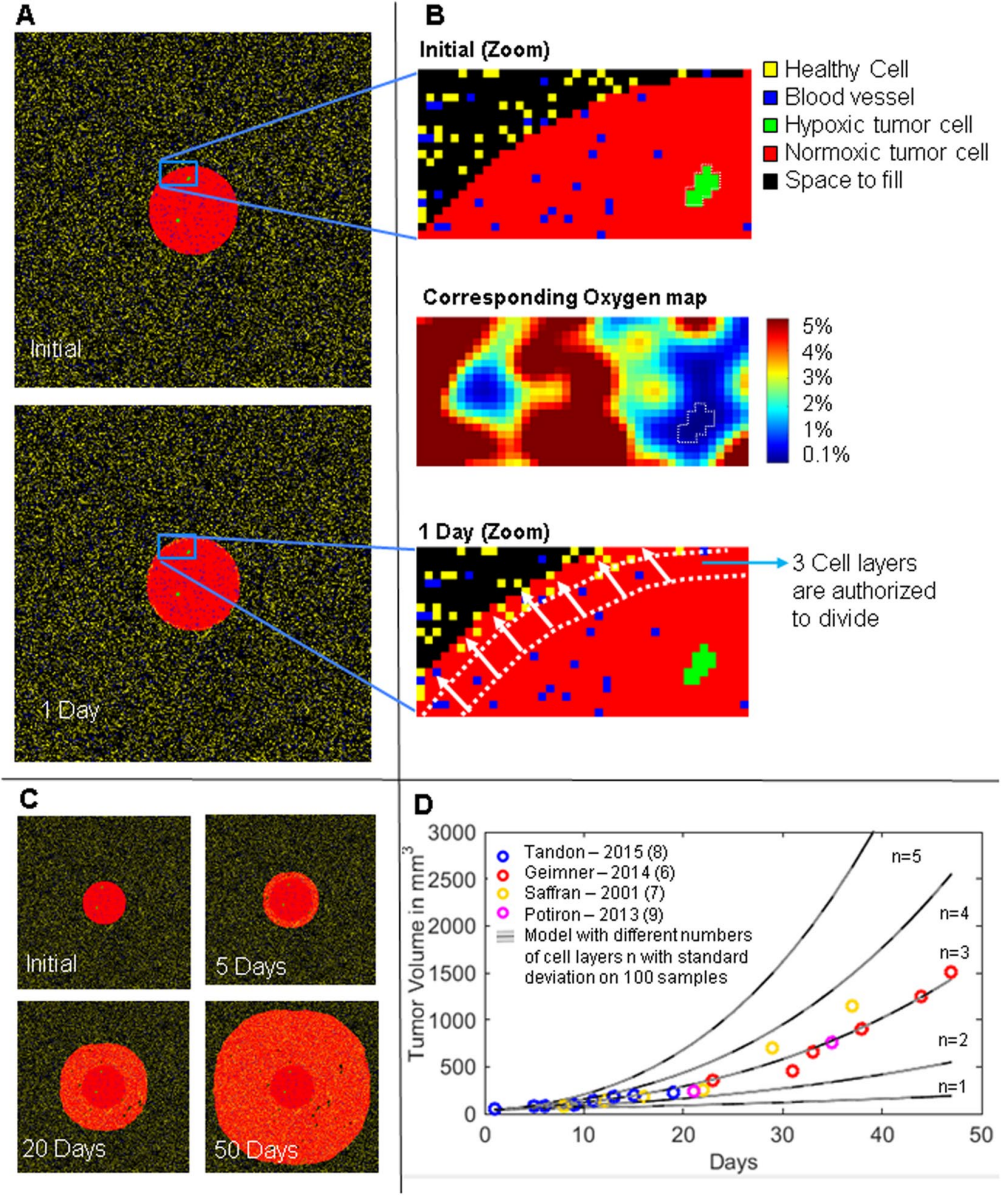
Tumour Growth Model. (**A**) Automata model of the tumour at day 0 and 1 day later, i.e. one cell cycle later. (**B**) Each pixel corresponds to a tumour and endothelial cell state. Endothelial distribution, i.e. vessel network, allows computation of an O2 ratio in the tumour map. (**C)** Tumour growth simulation without irradiation. (**D**) Calibration of the number of cell layers authorized to divide at the tumour periphery based on growth data obtained from the literature for PC3 tumours in mice. Setting cell layers to 3 gives the best fit for these data (i.e. n = 3).

As we have shown previously for normal breast tissue^6^, the tumour microenvironment is simulated as a matrix of pixels with integer values that represent either a cell type, an open space or a blood vessel. For each time iteration, the status of all pixels is updated based on biological rules, allowing us to take advantages of an image processing algorithm to model complex spatial events such as oxygen (O2) diffusion from blood vessels or cell division. The rules and states of the model are summarized in Supplementary Fig. S1. Note that all image processing steps are performed using the advanced imaging MATLAB toolbox DIPimage (free non-commercial license, Delft University of Technology, Delft, The Netherlands). *In silico* experiments consists of a 2D array of 600 × 600 pixels for the tumour and peritumoral tissue, with an extra dimension corresponding to the number of simulated days (i.e. one time step per day, and the number of steps representing the duration of the radiation protocol and the extra weeks for monitoring recurrence). Figure 1A illustrates the original tumour at the beginning of treatment: tumour cells can be either located in an area where O2 levels are above 0.2% (normoxic region - cells shown as red pixels and labelled as type 1), or below 0.2% (hypoxic regions - green pixels, labelled as type 2). The threshold is used only to provide visual feedback on the hypoxic regions: the actual O2 level at each pixel position is taken into account when computing radiation effects, as described in subsequent sections. The original tumour before treatment is a 1.5 mm diameter (100 pixels) disk, composed of square cells of 15 µm side for a total of 7845 cells inside the entire tumour allowing time-relevant simulations, and equivalence to simulations of a 3D tumour as illustrated in Supplementary Fig. S2. The surrounding tissue is composed of 20% of quiescent normal cells (type 3 – yellow pixels) and the rest is accessible space for tumour cells to grow in. Simulating blood vessels is essential in this work and each vessel is simplified as a single type 4 pixel (dark blue pixels in Fig. 1B) crossing the 2D tissue perpendicularly at random. In reality vessels are larger than one single cell. Nevertheless, capillaries are typically of 5 to 10 micron radius, and these values are often used in modelling work^7–9^. We will also show subsequently that such simplification is accurate enough for modelling O2 diffusion from blood vessels to tissue. Oxygen maps are computed using simple diffusion law and O2 maps are updated for each time iteration step as illustrated in Fig. 1B.

Figure 1(B–D) Also illustrate how the automata rules imposed in this model (see Supplementary Fig. S1) dictate tumour growth. Briefly, tumour cells can only divide if space in neighbouring pixels is available (i.e. contain no cell). Therefore, in the 2D tumour disk model, only tumour cells on the outer layer of the disk can divide. In order to determine the main parameters of tumour growth in our model (Supplementary Table S1) we need *in vivo* data to benchmark our simulations. A well-defined human prostate tumour cell line, PC3, has been grown as xenograft in mice^10–12^ or orthotopically in our lab^5^. Tumour growth curves from these various studies may thus be compiled into one dataset by allowing the time reference for each individual experiment to be shifted so that tumour volume would match the same time points. The resulting data points are shown in Fig. 1D. In addition, each time step can be derived experimentally and is set to 24 hours based on the reported average cell cycle time of PC3 cells^13, 14^. We first test the model by assuming that only the outer cell layer is dividing. Note that to compare simulated 2D growth curves to *in vivo* 3D volume, we had to scale the cell number N at each time point so that the simulated disk represents the cross-section of a full tumour. The spherical tumour volume can then be approximated as *V*_*e*_ in the following manner:

1 $$\pi {r}^{2}\,\underline{\underline{{\rm{m}}}}\,{s}_{c}N$$

2 $$r\,\underline{\underline{{\rm{m}}}}\,\sqrt{\frac{{s}_{c}N}{\pi }}$$

3 $${V}_{e}(N)=\frac{4}{3}\pi {r}^{3}=\frac{4}{3\sqrt{\pi }}({{s}_{c}}^{3/2})({N}^{3/2})$$

where *r* is the radius of the simulated disk, *N* the cell number for one time point in the 2D simulated tumour and *s*_*c*_ the average area of one tumour cell derived experimentally from microscopic sections^15^ (i.e. 15 × 15 µm^2^).

Resulting growth curves are slower than the compiled *in vivo* data (Fig. 1D, cell layer division n = 1).

We hypothesize that this reflects the fact that tumour cells move from the inside to the outside of the tumour^16, 17^ and non-surface cells may also have an opportunity to divide. We therefore need to model the impact of cell movement on the tumour growth rate while trying to keep the model as simple as possible by not having to track individual cell movement. This is done by introducing the notion of “available space”, which authorizes cells more than one layer away from the outer boundary of the tumour to also divide. The implementation of “n cell layers division” is performed iteratively using an image-based algorithm to reproduce the spatial growth limitation observed in real tumour. We believe that this approach produces more realistic behaviour than classic deterministic approaches which assume homogenous geometries.

The imaging process is performed in multiple steps. First, non-dividing cells are identified by applying a binary erosion on the tumour mask with n iterations, resulting in a smaller mask of cells which are too deep inside the tumour to divide (i.e. the resulting mask is more than n inside layers deep). Similarly, we define the area where new cells can occupy space (the growth area) by applying a binary dilation with n iterations on the tumour mask. Pixels of type 3 and 4 (normal cells and blood vessels respectively) are excluded from the growth area which restricts the amount of space available. Next, the outermost layer of tumour cells is duplicated first by randomly filling the growth area with the same number of cells present in the first layer. We repeat this process iteratively for the second and third layers until the n^th^ inside layer, each time making sure that the new number of cells is less than or equal to the number of cells in the layer being duplicated, and less than or equal to the number of free pixels remaining. Note that if too many healthy cells occupy the surrounding space around the tumour, not all cells from the deeper layers have enough space to duplicate. We initially set 20% of the space surrounding the tumour to be occupied by healthy cells, based on experimental observations. This parameter is somewhat arbitrary and one can get the same tumour growth curve by increasing simultaneously the percentage of healthy cells with the number of dividing layers (data not shown). This parameter is therefore not modified further in this work and only the number of dividing layers is investigated by parameter sweep. Figure 1D suggests that n = 3 leads to the most accurate tumour growth prediction compared to the compiled *in vivo* data^5, 10–12^. Therefore, n = 3 is used thereafter for all subsequent simulations involving radiation. However tumour growth has been shown to follow a sigmoidal pattern in both avascular and vascular tumour^18^: the model we used here may not hold for larger tumour sizes, where a plateau of growth will be reached. Our model in its actual state would then have to be considered only for tumour reaching sizes up to the inflection point of the sigmoid.

### Modelling steady state oxygen concentrations in tumour

Our tumour model incorporates the O2 diffusion through blood vessels assuming that the oxygen consumption of tumour cells is steady (i.e. the tumour is in homeostasis leading to no net change of oxygen from cell consumption). O2 maps, as illustrated in Fig. 1B, are computed for the tissue at each time step and they report the O2 amount for any cell *c* (i.e. pixel) of the tumour or healthy tissue (noted *O*_2_(*c*)).

**Figure 2:**
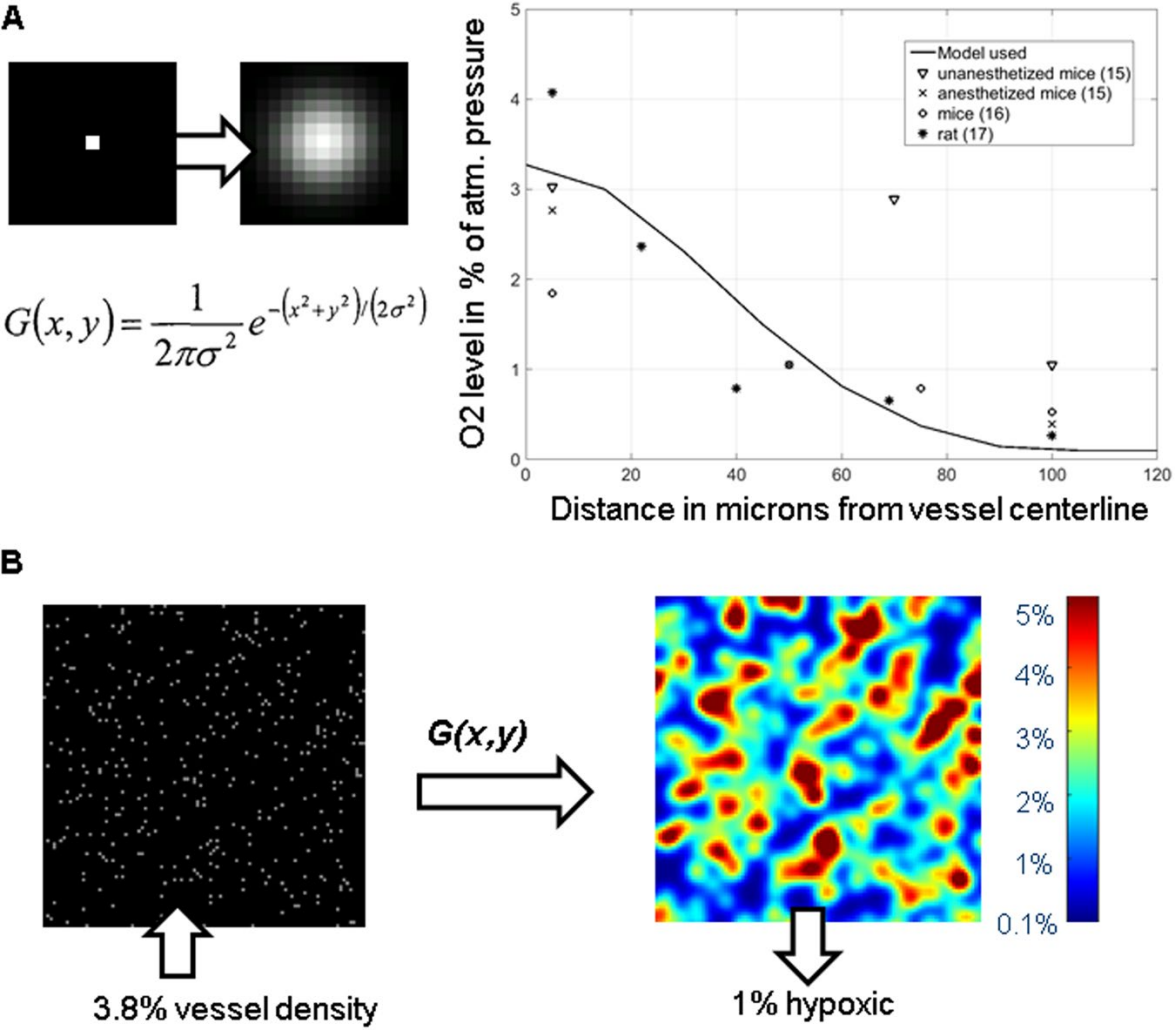
Modeling steady state oxygen concentrations in tumour. (**A**) The diffusion process is modeled as a Gaussian filter applied on the vessel map. The coefficient of diffusion of the filter was calibrated on experimental data of the partial pressure of O2 in mmHg against the distance from one vessel wall on colon and mesentery. (**B**) D (diffusion coefficient of O2 from the vessel) was fixed in a plausible range to fit experimental data to a vessel density at 3.8% and checking D values for which the percentage of hypoxia area (threshold at 0.2% in the O2 map) was measured experimentally below 1%^55^, while conserving an average level of O2 in the tumour of about 3%.

As illustrated in Fig. 2, *O*_2_(*c*) is computed by applying a spatial Gaussian filter to a Boolean mask defined by all pixels of type 4 (blood vessels).

First, the sigma value for the Gaussian filter is derived by fitting the O2 profile measured radially away from one single blood vessel using the diffusion equation:

4 $${O}_{2}(x)=s\ast \exp (\frac{-{x}^{2}}{2{\rm{D}}})$$

The fitting gives a range of plausible values for D (sigma squared of the Gaussian and diffusion coefficient from the vessel) and s (the scaling factor of O2 concentration) with a 95% confidence interval. We get a fit value of sigma of 2.7 with a confidence interval of 1.6 to 3.7, assuming a pixel size of 15 µm. The data for the model are derived from measurements made of the partial pressure of O2 in mm Hg in anesthetized and un-anesthetized mice and rats^19–21^ after converting pressure to percentage of the atmospheric pressure at sea level, which is 760 mm Hg (i.e. 21% in ambient air).

Next, we estimate the density of blood vessels from tumours sections using immunohistochemistry, which shows that 3.8% of the space is occupied by blood vessels (Supplementary Fig. S3). We then apply known tumour oxygenation levels. Our previous work found that only 1% of tumour cells are hypoxic at the beginning of the treatment^5^; other studies report average O2 concentrations of around 3%^15, 22^. We search in the space of simulated oxygenation levels, generated from the O2 maps, which are dependent on the range of plausible values of D (sigma) and on the blood vessel density; the value of D allows the two oxygen conditions (hypoxia and average O2 concentration) to be filled. Figure 2B shows the corresponding O2 map matching O2 levels in tumour data. Note that some regions of the tissue reach the maximum authorized concentrations of 5%, corresponding to complete O2 saturation^22^. By fixing the blood vessel density (type 4 objects) crossing the 2D tumour perpendicularly at 3.8%, only one value from the plausible range of D (coefficient of diffusion D = 5.76 pixels corresponding to a sigma of 2.4) fulfilled both O2 conditions, and this was used in all simulations.

### Modelling radiation effect on endothelial cells

Two types of damage from ionizing radiation have been reported for blood vessels. In conventional dose ranges (see Supplementary Table S2), we have observed that blood vessels become more permeable and increase tumour oxygenation after protracted dosing5. At doses above 6 Gy, radiation induce vessel death^23^, which should increase hypoxia and tumour radioresistance, a phenomenon known as the tumour bed effect^24^. These two blood vessel responses lead to opposing effects on tumour oxygenation during treatment. As described below, we introduce simple mathematical formalisms for each of these processes to evaluate their relative impact on tumour control.

The increased O2 permeability from blood vessel following protracted doses^5^ is poorly understood but we can at least make some unambiguous assumptions. We hypothesize that such phenomena are caused by individual cell death in the blood vessel, which compromises the vessel. We therefore anticipate dose dependence and we propose to use classic cell death measured in human endothelial cells^25^ as a first approximation for blood vessel disruption. This is implemented in automata by the classic linear quadratic model^26, 27^ and Monte Carlo approaches. In our 2D tumour model, whenever a vessel is exposed to a given dose fraction *fracD*, we compute the probability of losing one cell as the hit probability, where *α*_*ec*_ ${\alpha }_{ec}$ and *β*_*ec*_ have been computed from the experimental data in ref. 25:

5 $${P}_{v\_hit}(fracD)=1-\exp (-{\alpha }_{ec}\,fracD-{\beta }_{ec}\,frac{D}^{2})$$

**Figure 3:**
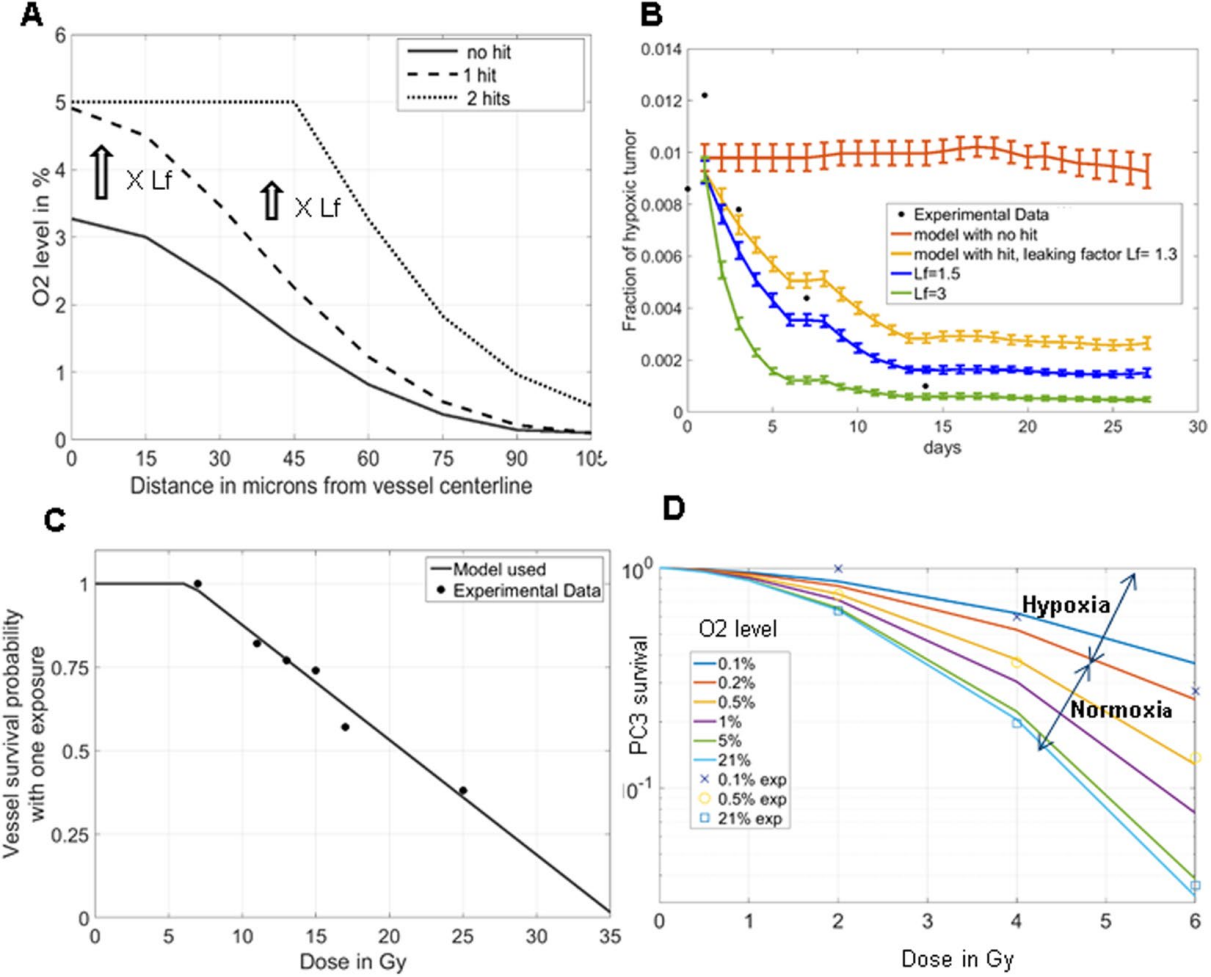
Modeling the impact of oxygen on radiotherapy. (**A**) Previously published model of O2 “leakage”^5^ derived from a Monte Carlo approach with a probability of “hitting” a vessel after a given dose defined by the corresponding cell death probability of endothelial cells measured *in vitro*. If there is a “hit” to a blood cell, a multiplicative factor *Lf* is applied to the profile. (**B**) Determination of *Lf* based on the experimental evolution of the fraction of hypoxic tumour over time: leakiness of 50% (leaking factor of 1.5) led to most accurate prediction of the published^5^ fraction of hypoxic surface drop (blue curve versus circles). (**C**) Model of the vessel survival probability against a single dose, fitted from previously published experimental data^23^. (**D**) **HRF** « hypoxia reduction factor » was fitted using a Howard-Flanders fit based on our previous published data^5^ (See Fig. S4). Survival curves of tumour cells can then be predicted for various O2 levels using the fitted HRF. Tumour cell death at full oxygenation is based on the PC3 LQ data: alpha = 0.0441, beta = 0.0898. The predictive curves accurately overlap previous experimental points.

If a vessel has been “hit”, then we assume the amount of O2 passing through the endothelial wall is increased by a fixed multiplicative “leak factor” *L*_*f*_, while actual O2 diffusion does not change (i.e. the sigma of the Gaussian filter remains unchanged). This process is illustrated in Fig. 3A. As in the previous section, O2 levels saturate at 5% so any O2 value above 5% is capped at 5%.

In order to evaluate possible values for *L*_*f*_, we start with a value of 1 (i.e. no effect) and increase it incrementally until we reproduce *in vivo* measurements of the fraction of hypoxic cells in the tumour following the protracted dose experiment we conducted in the past^5^. *L*_*f*_ values between 1.3 and 1.7 lead to reasonable fit and we used *L*_*f*_ equal to 1.5 for the rest of this work (corresponding to the predicted profile shown in Fig. 3B).

Radiation-induced blood vessel death and loss of oxygenation has been previously reported^23^ and we simply model it using a death probability that is linear with dose with a threshold at 6 Gy and 100% cell death at 35 Gy (Fig. 3C). We also assume that multiple protracted doses are not synergistic on vessel death: i.e. damage leading to full vessel death is not cumulative, an assumption that cannot be confirmed by experimental data at this time. More generally, both increased perfusion and vessel loss should be further characterized *in vivo* to create a more accurate model that fully describes the impact of repeated dose exposures on the integrity and fate of blood vessel function.

### Modelling radiation effect on tumour cells

This section describes how ionizing radiation, inducing cell cycle arrest and cell death, was modelled.

Radiation-induced growth arrest describes cells which are blocked in G1 or G2 for a duration dependent on the acute dose. Experimental data on cell growth arrest for prostate tumours are limited. We have previously described the effect of a single dose (4 Gy) at 36 hours^15^. We therefore adopt the simplest model, where cell growth duration is proportional with dose. The single dose measurement can then be used to determine the slope (see Supplementary Table S1 and Supplementary Fig. S5). We will discuss the importance of this missing information later.

Secondly, radiation-induced cell death has been the focus of radiation oncology for many decades and is well understood and characterized. For instance, the survival probability of tumour cells after irradiation is traditionally calculated by fitting clonogenic survival data using the linear quadratic (LQ) model^26, 27^ given by:

6 $$S(fracD)=\exp (-\alpha fracD-\beta frac{D}^{2})$$

*In vitro* cells are typically cultured under 21% O2. However, this scenario differs from natural conditions *in vivo*. For most tissues, O2 concentrations vary from 2 to 5% and may fall to less than 1% in tumours^22^. O2 levels modulate clonogenic survival. For this reason, the O2 enhancement ratio (OER) was introduced in radiotherapy and refers to the enhancement of therapeutic or detrimental effect of ionizing radiation in presence of O2^28^. Another way to characterize OER is by the hypoxia reduction factor (HRF) which is the ratio of the doses for a specific iso-effect under a given oxygenation condition compared against the condition of 1 Gy at 21% O2^29^. In order to predict cell death for a given cell c in the tumour, we apply the LQ fit of the clonogenic survival at a dose corrected for the O2 level ${O}_{2}(c)$ using the HRF. This is done by first fitting the experimental data for HRF in PC3 cells^15^ as shown in Supplementary Fig. S4, using the Howard-Flanders mathematical formalism^30^ as described in ref. 29:

7 $$HRF(c)=\frac{mK+{O}_{2}(c)}{K+{O}_{2}(c)}$$

where *m* is the maximum *HRF* and *K* is the oxygen partial pressure at which the *HRF* is half the maximum value. The fitting with initial condition at 2.7 for m and 0.002 for K leads to the values m = 2.804 and K = 0.001076, as described in Supplementary Table 1, which lists all parameters.

Cell survival for each dose fraction *fracD* is then:

8 $$S(fracD)=\exp (-\alpha {D}_{eq}-\beta {D}_{eq}^{2})\,{\rm{where}}\,{D}_{eq}(c)=\frac{fracD\,}{HRF(c)}$$

This model leads to accurate prediction of cell death for the various calibrated doses and O2 levels as shown in Fig. 3D. Implementation of cell death in the simulated tumour is then based on Monte Carlo approaches. Briefly, at each time step involving a radiation exposure, the probability of death is computed based on the dose and O2 level at the location of the cell. This probability is compared to a random number between 0 and 1. If the random number is below the probability, the cell is condemned to mitotic death (cyan cells labelled as type 5).

## Results

### Radiation-induced cell death by mitotic catastrophe

To summarize material and methods, our model has so far incorporated the following assumptions:

1. Tumour cells have blood vessels intercalated with an O2 leakiness parameter;
2. Radiation can make blood vessels leakier for O2;
3. Conversely, high radiation doses can disable a vessel’s capacity to perfuse O2;
4. Tumour cell death is derived from *in vitro* clonogenic survival modulated by O2 concentrations.
5. Tumour cells can grow as long as there is free space around them.
6. All tumour cells are growth-arrested when exposed to ionizing radiation and the duration of the arrest is proportional to the dose received.
7. Tumour cells behaviour does not change with repetitive exposure (no adaptation).

There is one last aspect of the model that deserves more attention regarding the translation of *in vitro* clonogenic survival data to the actual death of the tumour *in vivo*. The clonogenic survival assay in mammalian cells was first developed by Puck and Marcus in 1956^31, 32^ and has been the basis of the fractionation protocol for standard radiotherapy for the last 50 years. However, the data derived from such measurements is insufficient to inform our computer model. First, we cannot differentiate the different types of cell death. For example, apoptosis and mitotic death – where a cell actually disappears from the tumour– cannot be modelled in the same way as senescence – where the cell remains inside the tumour but can no longer divide. For now, we will ignore senescence. In addition, we do not know how long it will take for a cell with a lethal damage (i.e. type 5) to be cleared from the tumour. Death and cell clearing is not instantaneous after exposure and tumours undergoing radiotherapy do not shrink at the rate one would expect from measured *in vitro* cell death. Recent clonogenic survival data on lung cancer cells have in fact shown that it can take as long as 14 days post-exposure before a cell actually disappears^33^. Similarly, we have shown using time lapse imaging of normal human breast cells *in vitro* ^34^ that cell death is asynchronous following exposure to the same dose of x-rays with large variations between cells.

In our model, we introduce the concept of “mitotic death” as the main mechanism of radiation-induced tumour cell death. This concept implies that a cell which has been marked with a lethal damage (type 5 – cyan colour) will be removed from the simulation only when it undergoes division upon availability of space. Supplementary Fig. 1 illustrates the flow chart for implementing mitotic catastrophe. This simple mechanism is in good agreement with the general consensus of how mitotic catastrophe occurs^35^ and it circumvents the need to introduce another parameter required for the cell death delay model that we and others have previously used^6, 33^. It also neglects senescence and apoptosis as other mechanisms for radiation-induced tumour cell death.

**Figure 4:**
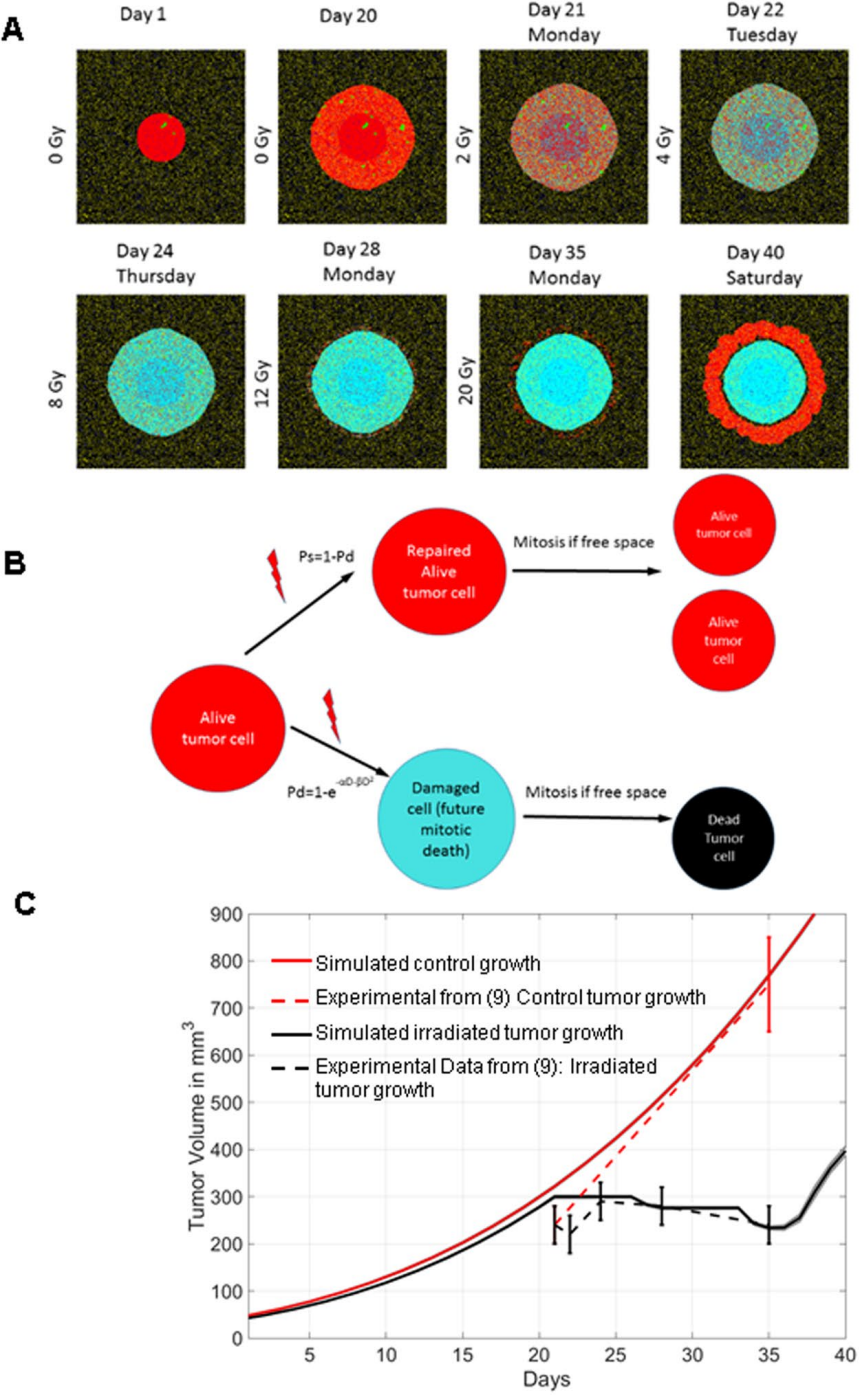
Implementation of mitotic catastrophe with automata. (**A**) Snapshots of simulation for tumour growth being treated after 20 days of growth unchallenged by ionizing radiation. Treatment starts on day 21 and consists of 2 Gy daily for two weeks, excluding weekends. Red cells are tumour cells located in normoxic regions, green cells are tumour cells in hypoxic regions. Once treatment starts, lethally damaged cells are labeled cyan and will be removed from simulation once they reenter cell cycle (i.e. mitotic catastrophe). (**B**) Simple flow chart describing how cells with lethal damages are labelled using the clonogenic survival fit obtained *in vitro* in the context of mitotic catastrophe. (**C**) Comparison of experimental data^5^ with simulations. 10 doses of 2 Gy (black dashed line with standard deviation of measurements) vs. control growth (Red dashed line with standard deviation). Simulated tumour volume for control growth (red line, no irradiation) and for irradiated tumour (black line), simulating the same protocol (2 Gy daily except weekend for two weeks), was computed from the number of cells when exposure began at day 21 to start at the same initial volume. The volume was extrapolated from cell number by taking an approximation of a spherical tumour, and then extrapolated from the disk surface, which is about proportional to the number of cells, by applying a corrective factor (see material and methods).

The implementation of mitotic catastrophe reveals some interesting tumour behaviour when simulating the response to a conventional radiotherapy. Figure 4 reproduces an experiment conducted in our lab where PC3 tumours were first transplanted orthotopically into an NMRI nude mouse and left to grow unchallenged for 20 days before being exposed to the daily exposure of 2 Gy. Irradiation was stopped after delivering a total dose of 20 Gy. The computer model parameters were set to reproduce these conditions. The predicted outcome is shown in Fig. 4A with snapshots of simulations throughout the 40 day observation period from tumour transplantation. Figure 4B illustrates the logical diagram for mitotic death. Tumour overall growth prediction matches experimental data extremely well as shown in Fig. 4C. Closer examination of the snapshots from Fig. 4A, simulations reveals interesting emerging properties of the tumour response to radiation. First, as long as the tumour is being irradiated daily, it stops growing and the number of cells programmed to die after division (type 5 cyan) continuously increases with each fraction. Second, whenever the tumour is left unchallenged over the weekend, the outer layer of the tumour re-enters cell cycle leading to the actual removal of this layer due to considerable mitotic catastrophe. However, one can also observe that re-entering the cell-cycle during the weekend leads to expansion of viable cells which had not been lethally hit during the week (sparse red cells in outer layer). Finally, the model reveals that tumour cells located deep within the core of the tumour have no opportunity to divide during the weekend and will eventually receive a lethal event from the repeated daily exposure in subsequent weeks of treatment. In this experiment, the model predicts that stopping treatment after only 20 Gy with 2 Gy daily will lead to tumour recurrence, as was observed experimentally (Fig. 4C). Note that one important parameter in this model is the duration of cell-cycle arrest which determines wether or not the tumour will have time to re-enter the cycle during the weekend. It is currently set at 36 hours following 4 Gy^5^ and changing this value will have important effect on simulations (Supplementary Fig. S5).

### Determining tumour control probability

**Figure 5:**
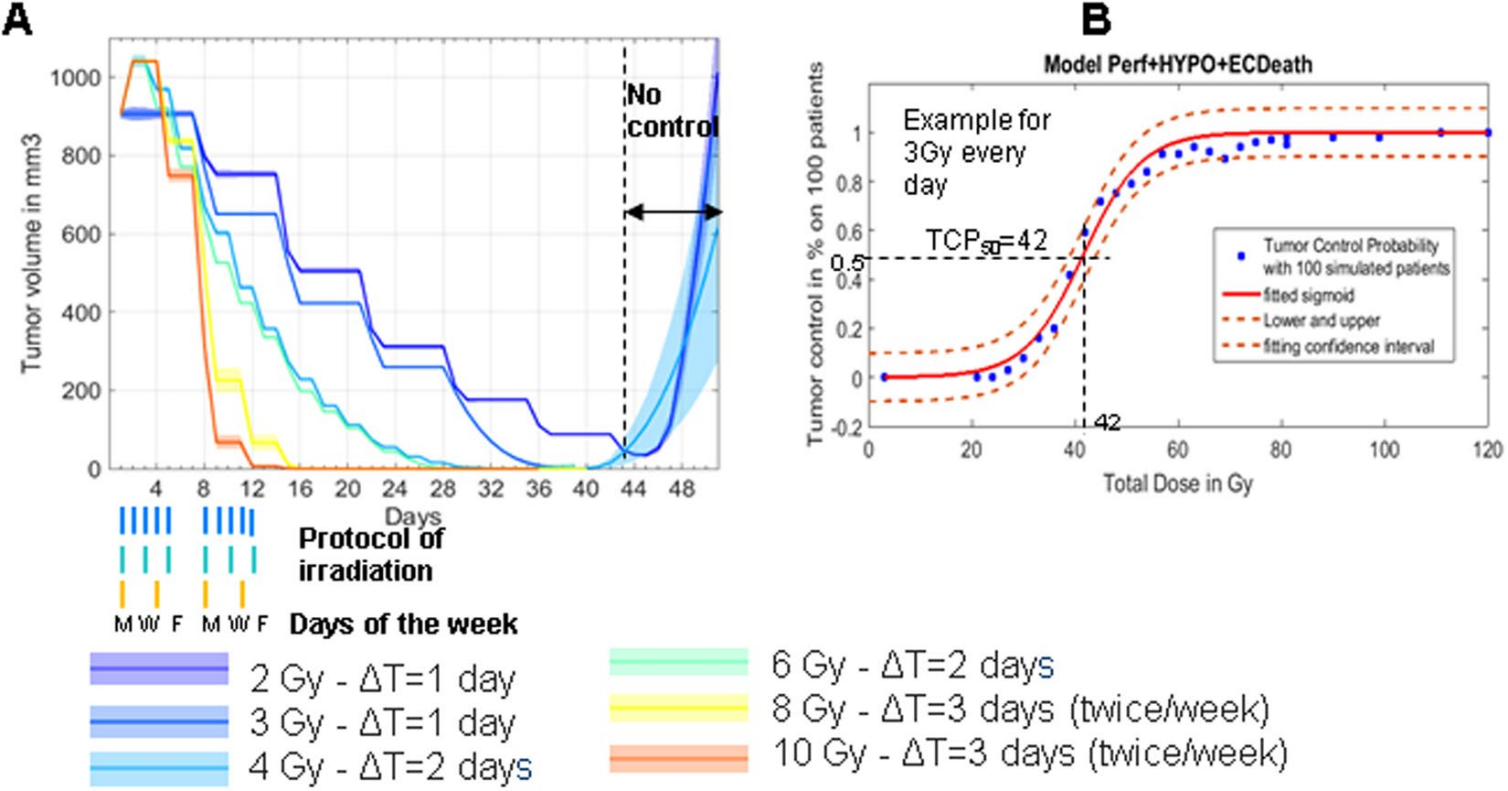
Determining Tumour Control Probability. (**A**) Simulated tumour volume in mm^3^ for 100 “*in silico* mice” as a function of day, all with a total delivered dose of 60 Gy (56 Gy for individual dose of 8 Gy, 7 irradiations). The time interval between each exposure is indicated in days as ΔT. The dose for each fraction is given in Gy. For example, 2 Gy daily for 6 weeks (purple line) does not achieve any tumour control: the majority of animals have recurring tumours at day 44. Conversely, at 3 Gy daily (blue line), 100% tumour control was obtained with a total dose of 60 Gy. (**B**) Tumour control curves for the 3 Gy daily protocol. The average % of Tumour killed for a given total dose fits a sigmoid curve. Because each fraction is 3 Gy, average points are spaced by 3 Gy interval. The fit suggests that a dose of 42 Gy would lead to 50% of mice cured from the tumour. A dose of 60 Gy (i.e. 20 fractions) would suggest 100% control.

After validating the model on experimental data, we can now use the model to generate predictions for various radiation protocols and visualize the impact of the different biological mechanisms implemented in this model: i.e. tumour oxygenation, radiation-induced perfusion and radiation-induced vessel death enhancing hypoxia. To better visualize such impact, we monitor the volume of tumours for various radiation protocols (Fig. 5A). We define “tumour control” in the model as the condition in which there are no more cell of type 1 or 2 (i.e. viable tumour cells) at the end of the treatment.

Using this definition, tumour control probability (TCP) curves are generated by simulating 100 “*in-silico* mice” for each protocol of interest, with different “seeds” for each set of random numbers used for probability assessment in our Monte Carlo approach. Different clinical protocols are tested, with one constant dose per fraction and one constant time interval between each exposure. It is assumed that no treatment takes place at weekends. After each additional dose fraction, each animal simulation is evaluated to test if there are any remaining type 1 and 2 cells. If not, the tumour is considered controlled and after each fraction, the TCP is updated as the ratio of controlled tumours within 100 simulated tumours.

The resulting simulated TCPs have the well-known sigmoid shape which can be fitted using the sigmoid equation (Fig. 5B):

10 $${\rm{TCP}}({D}_{t})=\frac{1}{1+\exp (-\lambda {D}_{t}+\delta )}$$

where ${D}_{t}$ is the total dose received following one particular protocol, $\delta \,\,$takes into account the shift in the inflexion point of the sigmoid, and $\lambda$ describes the tangent at the inflexion point.

The total dose *D*_*t*_ for one particular protocol leading to a TCP of 50% (0.5 on the sigmoid), i.e a 50% probability of achieving tumour control using a particular protocol) is computed by solving the previous equation with the fitted parameters such that TCP(*D*_*t*_) = 50. The lower and upper 95% confidence interval of the TCPs are also computed by measuring the observation bounds, taking into account the uncertainty in the fitting, and the random variations in the observations. The error on the evaluation of the TCP of 50% is then directly derived from the confidence bounds on the TCP of this dose.

### Characterizing the impact of vascular disruption on radiotherapy efficacy

**Figure 6:**
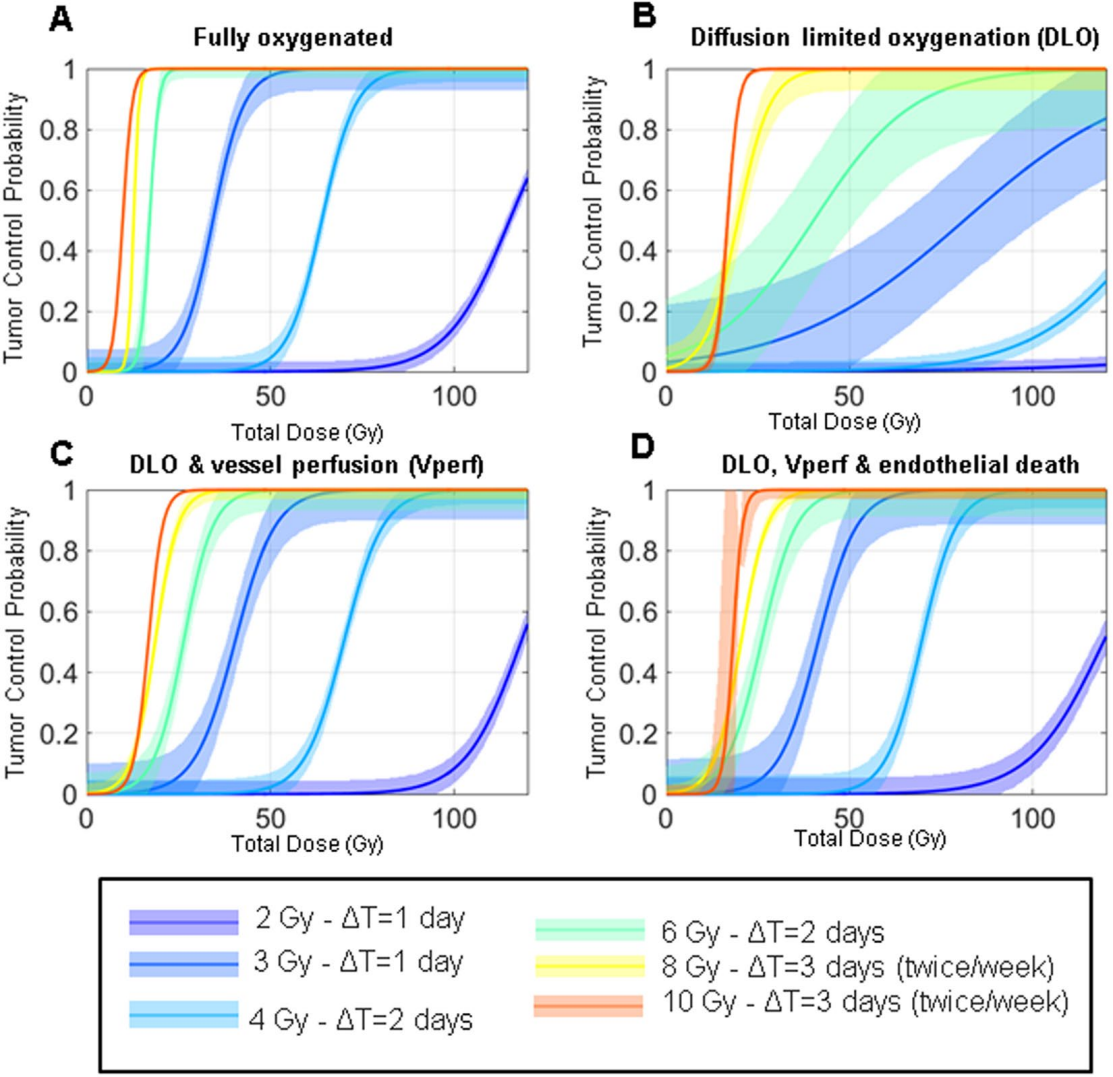
Tumour control probability vs dose/protocol. Sigmoid fits of TCP with 95% confidence interval are shown for various protocols. Simulations using the standard LQ model when tumour is (**A**) fully oxygenated (O2: 21%); (**B**) O2 diffusion is limited (O2: <5%; Diffusion limited oxygenation (DLO)); (**C**) DLO and irradiation-induced vessel perfusion (Vperf); (**D**) DLO, Vperf and hypoxia driven by irradiation-induced endothelial cell death.

**Figure 7:**
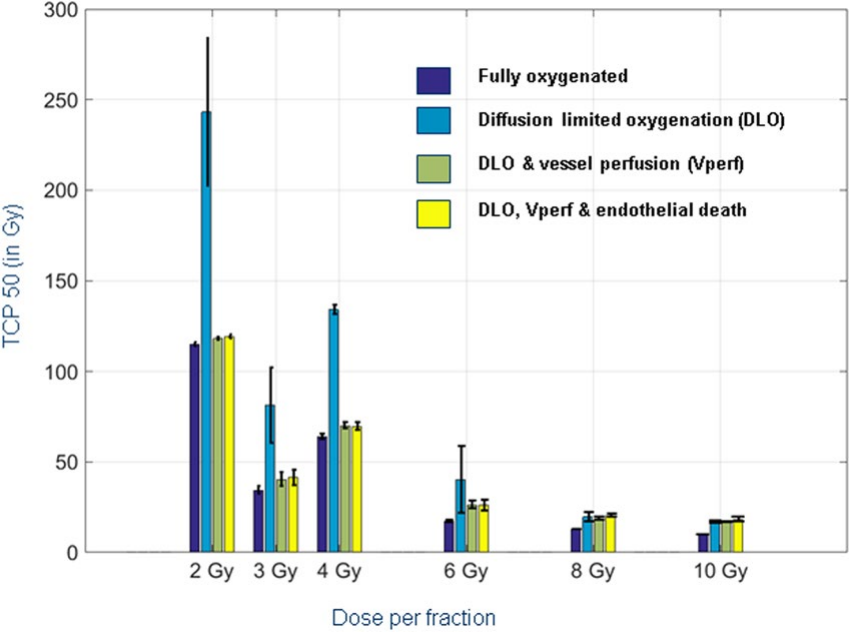
Visualizing the impact of various vessel damage models on TCP50. Each panel compares the total dose (Gy) required to reach the TCP50 for each given protocols (see Fig. 6).

Fitting of TCPs obtained for dose fractionation mimicking clinically relevant protocols are shown in Fig. 6. All the tested clinical protocols are described in Supplementary Table S2 and readers can test their own protocol using the provided supplementary software. TCPs for the same protocols are shown in four different panels, each representing simulations for different biological assumptions impacting endothelial behaviours and O2 rate in the tumour. Figure 6A assumes full oxygenation in the tumour (i.e. clonogenic death at 21%), while Fig. 6B takes into account reduced O2 levels in the tumour but without perturbation of O2 diffusion from repetitive exposures. On Fig. 6C, the biological assumption of vessel perfusion is added, partly counterbalancing the effects of hypoxia. On Fig. 6D, the death of vessel from repetitive exposure is added increasing hypoxic regions as shown in Supplementary Fig. S6. To better visualize the impact of each biological assumptions, TCP50 are also graphed for each protocol and biological assumption in Fig. 7.

First, as expected, TCP50 decreases with the dose fraction used. Secondly, results show that neglecting the low oxygenation of the tumour environment lead to under estimation of the dose required to fully control the tumour (i.e. Fig. 6A shows that fully oxygenated tumour reaches 100% control at lower doses than in Fig. 6B where oxygen diffusion is limited). In addition, Figs 6 and 7 also show the importance of increased blood vessel perfusion during repetitive exposure. Without it, hypoxia is extremely high in the tumour leading to poor tumour control (Fig. 6B and cyan bars in Fig. 7). Perfusion alone brings back tumour control to lower doses, but TCP50s are still higher than if hypoxia is not considered at all. It is important to note that the vascular perfusion counteracts the hypoxia radiation resistance better at lower dose fraction in our model. This probably reflects the fact that as the dose fraction increases, the number of fractions to reach TCP50 drops. For example, in the case of hypofractionation (≥8 Gy per fraction), TCP50 is reached with only three or fewer fractions minimizing the impact of blood vessel perturbation which cannot affect the first exposure.

The last biological assumption simulated is the vessel death from high doses (occurring only at ≥ 8 Gy per fraction). When blood vessel death is included, hypoxia does not drop severely anymore with 8 Gy fractions (black solid line curves in Fig. S6) and is even significantly increased at 10 Gy. We would therefore hypothesize that the optimum dose for hypofractionation protocols should be between 8 and 10 Gy. However, as seen in Figs 6D and 7, endothelial cell death does not modify TCP50 for any dose fractions. This lack of effect is again likely due to the high tumour cell death at high dose, leading to a strong tumour control from the first dose as described in the previous paragraph for perfusion.

## Discussion

Different modelling approaches have been proposed in the literature for radiotherapy. In 1989 Fowler *et al*.^26^ introduced the term BED, biologically effective dose, as a linear quadratic (LQ)-based formula with an overall time factor and a tumour repopulation time delay during fractionated radiotherapy courses. This formalism is used almost universally for calculating isoeffect doses of different fractionation schemes and is therefore of fundamental relevance in daily clinical practice^26, 36^. However, the applicability of this model to high dose per fraction is controversial^37, 38^. This radiobiological debate has become increasingly relevant due to the widespread adoption of stereotactic body radiation therapy (SBRT), where irradiation is delivered in a few fractions or even in a single fraction of very high dose^39^. Last decade, several models were proposed for SBRT therapeutic schemes, such as the modified Linear-Quadratic (MQL) model^40^, the Linear-Quadratic-Linear (LQL) model^41^, the generalized Linear-Quadratic (gLQ) model^42^ or the universal cell survival (UCS) curve^43^. In addition to those simple mathematical formalisms, complex stochastic models that include more realistic biological processes have been developed to take into account phenomena such as cell cycle phase, vascularization or hypoxia. In their reviews, Marcu *et al*.^44^ and Enderling *et al*.^45, 46^ presented models that simulate tumour growth and/or radiotherapy incorporating tumour oxygenation (i.e. vascularized tumours). These reviews concluded on the need for better description of biological processes to improve computer models in the field of radiation oncology. Emphasis on tumour oxygenation, tumour heterogeneity and cancer stem cells were noted as important factors in this modelling effort.

In the work presented here, we propose an approach which addresses the heterogeneity of the tumour oxygenation in a new way using cellular automata. However, unlike classical models of tumour growth^47^ where oxic rate is modelled with partial derivative equations, we use a simpler and faster formalism simulating diffusion processes via Gaussian image filtering. Other simpler oxygen models include the Krogh model assuming cylindrical diffusion^7, 48^ or even spherical diffusion kernels^9^. In addition, we introduce mitotic death as the main mechanism for radiation-induced death. And lastly, all the parameters of our model are kept to a minimum and have a physical or biological meaning that can be obtained experimentally (Supplementary Table 1). The biological data used in this work also benefit from our ability to grow human PC3 prostate tumours inside nude mice by injecting them orthotopically^5^. Even though such biological model is a simplification of the human case where the immune response of the host plays an important role in the overall tumour response, such data remain essential to the validation of the basic parameters and mechanisms of our automata before moving to more realistic situations. In addition, *in vivo* data from orthotopic human tumours are still a clear improvement from traditional *in vitro* measurements typically used to characterize the radiation response of human tumours *in silico*. It would eventually be important for hypo fractionated radiotherapy in humans to also include the immune response and investigate the “abscopal responses” now reported in several studies^49–51^, but immunology response is primarily involved in the relapse of tumour after irradiation. Such modelling is beyond the scope of this study, which is dedicated to acute tumour regression.

A carcinogenesis automata model previously developed^6^, was therefore modified to model the perturbation of blood vessels in the tumour during radiotherapy and to investigate its potential impact on tumour control. Because the dependence of tumour cell death as a function of oxygen level is well understood and was reported previously by our group^5^, such information was easily integrated and validated in our model. Clonogenic survival and HRF measured *in vitro* were also included in the model, assuming the majority of radiation-induced cell death was through mitotic catastrophe. It is interesting to note that to this day, no computer models have introduced the use of mitotic catastrophe to model cell death despite its major role in tumour control^52^. Doing so, we managed to reduce the number of parameters in our model and explain why tumour size does not shrink much during treatment: i.e. radiation-induced cell cycle blockage prevents mitosis between fraction and there is therefore no cell death. The model has led us to a new working hypothesis suggesting that tumour control can be achieved better if the tumour is kept out of cycle during the entire treatment. Part of this hypothesis is based on the assumption that tumour is fully growth arrested after each fraction and that the duration of cell cycle arrest is proportional to the fractionated dose. This is obviously too simplistic as we know from our own work^34^ that some cells continue to divide after exposure to relative large doses of ionizing radiation and each cell responds uniquely to radiation. This can be refined in the future by adding cell cycle distribution measured *in vitro*. In addition, tumour cells are typically defective for G1 cell cycle checkpoints limiting the cells to a blockage in G2/M after large doses of ionizing radiation. Cell cycle state will be added to the model in the future.

Another direction of improvement of our model would be to use more physically grounded approaches for the OER (or HRF) modelling and for its influence on alpha/beta modulations. For now, we have followed the approach described in Carlson *et al*.^29^. Considering the mechanistic simulation of OERs by Grimes *et al*.^53^, or the model of modulation of alpha/beta ration by Wenzl and Wilkens^54^, would be of interest.

With these caveats in mind, one can infer important rules that may improve cancer treatment. For instance, our simulations showed that weekends increase the number of tumour cells re-entering cycle which give them an opportunity to escape death. We would therefore recommend increasing the dose on Friday to further reduce proliferation during weekends. Simulation also shows that the core of the tumour is more easily controlled by repeated radiation fractions, whereas cells on the outer layers of the tumours are more likely to survive treatment. Therefore, a large dose for the first exposure may help to control the outer layer better. The inclusion of our data regarding biological response of blood vessels following repetitive irradiation, allows assessment of the impact of blood vessel maturation and increased blood vessel perfusion for various radiotherapy protocols in our model. The results suggest that the best strategy is to use 8 Gy fractions to maximize tumour oxygenation via radiation-induced perfusion while having minimum increased hypoxia due to blood vessel death. However, the impact of endothelial apoptosis on the tumour response was not fully characterized. For instance, in addition to blood vessel loss for doses larger than 8 Gy, it is also known that endothelial apoptosis can induce radio sensitization for doses above 15 Gy^23^ further complicating the very high dose response. For this reason, more work is necessary to better understand the biology behind cell-cell interactions between tumour and blood vessel as function of dose and time.

In terms of model refinements, it would be interesting to modify the automata model to link DNA repair kinetic and cell cycle arrest to mitotic catastrophe. One could envision a model, given the right *in vitro* data, where the cellular heterogeneity in cell-cycle arrest for tumours is linked to DNA repair, so that damaged cells which spend less time repairing in G2 have a higher death rate during mitosis^35^. This would involve the gathering of detailed *in vitro* measurements by tracking individual cells for many generations as we previously did for non-malignant human breast cells^34^. We have started an ambitious program between our laboratories to monitor mitotic catastrophe, growth arrest and proliferation as a function of dose, O2 concentrations and time interval between repetitive exposures. The automata will be constantly refined as this information is generated. By looking at various tumour cell lines and characterizing the integrity of their cell cycle checkpoint via genomic analysis, we will also be able to better model the variability in tumour response and hopefully better understand what makes a tumour resist or adapt to radiotherapy. Finally, one aspect of this model that will also require further development is to include the response of the surrounding healthy tissue. This will be critical to validate any fractionation protocol, since a protocol needs to maximize tumour control while minimizing toxicity. Thus, tumour specificity as well as patient’s radiation sensitivity will be an essential aspect of future work.

Once further developed, we believe these new modelling methods will provide a powerful tool for precision medicine in radiotherapy taking us into a new paradigm beyond the linear-quadratic model for clonogenic survival in radiation oncology, introduced some sixty years ago^31, 32^.

## Electronic supplementary material

Supplementary Tables and Figures

Supplementary Software
