# Supplementary material for: Optimizing radiotherapy protocols using computer automata to model tumour cell death as a function of oxygen diffusion processes
Source: Sci Rep. 2017 May 23;7:2280. doi: 10.1038/s41598-017-01757-6 (PMC5442104; doi:10.1038/s41598-017-01757-6)
Supplement: Supplementary file 1 — Supplementary Tables and Figures [file 41598_2017_1757_MOESM1_ESM.pdf]

Optimizing radiotherapy protocols using computer automata to model tumour cell death as a function of oxygen diffusion processes.

Perrine Paul-Gilloteaux[1], Vincent Potiron[2], Grégory Delpon[2,3], Stéphane Supiot[2,3], Sophie Chiavassa[2,3], François Paris[2,3*] and Sylvain V. Costes[4,5*]

1. Structure Fédérative de Recherche François Bonamy, Micropicell, CNRS, INSERM, Université de Nantes, Nantes, France.
2. CRCINA, INSERM, CNRS, Université de Nantes, France.
3. Institut de Cancérologie de l'Ouest, Saint-Herblain, F-44800, France.
4. Biosciences, Lawrence Berkeley National Laboratory, MS:977, Berkeley, CA, 94720, USA.
5. NASA Ames Research Center Moffett Blvd, Mountain View, CA 94035, USA

Correspondence and requests for materials should be addressed to F.P. (email: francois.paris@univ-nantes.fr) or S.V.C. (email: sylvain.v.costes@nasa.gov)

Supplementary information:

Supplemental Table 1 and Table 2

Supplemental Figures S1-S6

Supplemental Table 1: Parameters in Cell automata model

Parameter description	Value used in simulations	Source for value
Tumor description		
Initial Tumor size	100 cells in diameter	Arbitrary fixed
Average cell cycle duration	24 hours	(13,14)
Occupied cell space, express as the size of one pixel	15 micrometers	Observation from microscopic images on PC3 (12,15)
Number of cell layers seeing available space for division	3	Based on control tumor growth best fit of experimental data on PC3 (6,10-12)see figure 1D
Tumor environment and oxygenation		
Healthy cell density, leaving gaps for division	20% of free spaces	Observation from microscopic images on PC3 (12,15)
Vessel density in the tumor	3.79% of tumor	Calibrated on the average expected level of oxygen on the tumor and the max % of hypoxia as measured experimentally in (15)
Diffusion and scale of oxygen Gaussian diffusion	D=5.76 pixel (i.e sigma =2.4)<br>Scale=1.18	Calibrated by experimental data of oxygen levels measured radially away from one single blood vessel (19-21) (LS174T tumor of SCID mice, measured by phosphorescence quenching microscopy, unanesthetised or anesthetised). See Figure 2
Irradiation effects on tumor and endothelial cells		
Growth Arrest Duration after radiation (single dose)	In days per dose<br>See Figure S5	Measured in (15) for one dose, extrapolated for the other.
Leak factor of vessels (hits effect on diffusion): each time there is a death hit in blood vessel, this vessel become more leaky by this factor	1.5	Calibrated to reproduce in vivo measurements of the fraction of hypoxic cells in the tumor following the protracted dose experiment (12) see Figure 3B

Vessel hits probability against dose in Gy, described as LQ model with Monte Carlo approach	Alphaec=0.19, Betaec=0.039	Based on experimental data from (26)
Death probability against dose in Gy, described as LQ model	Alpha= 0.0441, Beta= 0.0898	based on clonogenic data on PC3 (12)
Hypoxia Reduction Factor (to decide equivalent dose and consequently death probability based on alpha beta), Howard-Flanders fitting	m=2.804, K= 0.001076	obtained by fitting experimental data for HRF in (15) where clonogenic survival was measured for PC3 see figure S4
Death probability of a vessel at high dose	See Figure 3	Extrapolated from Garcia Barros data (23)
Protocol of irradiations		
Clinical fractionation scheme		See Table S2
Number of days of observation after last irradiation	15 days	Arbitrary to ensure that growth arrest duration and subsequent mitotic death are taken into account

Supplemental Table 2: Overview of clinical fractionation scheme tested in the simulation

Dose / Fraction [Gy]	Fractions Schedule (Days of week)	Total Doses [Gy] simulated
2	Mon Tue Wed Thu Fri	2 to 250 Gy by step 2 Gy
3	Mon Tue Wed Thu Fri	3 to 120 Gy by step 3 Gy
4	Mon Wed Fri	4 to 120 Gy by step 4 Gy
6	Mon Wed Fri	6 to 120 Gy by step 6 Gy
8	Mon Thu	8 to 80 Gy by step 8Gy
10	Mon Thu	10 to 80 Gy by step 10Gy

Supplementary figures

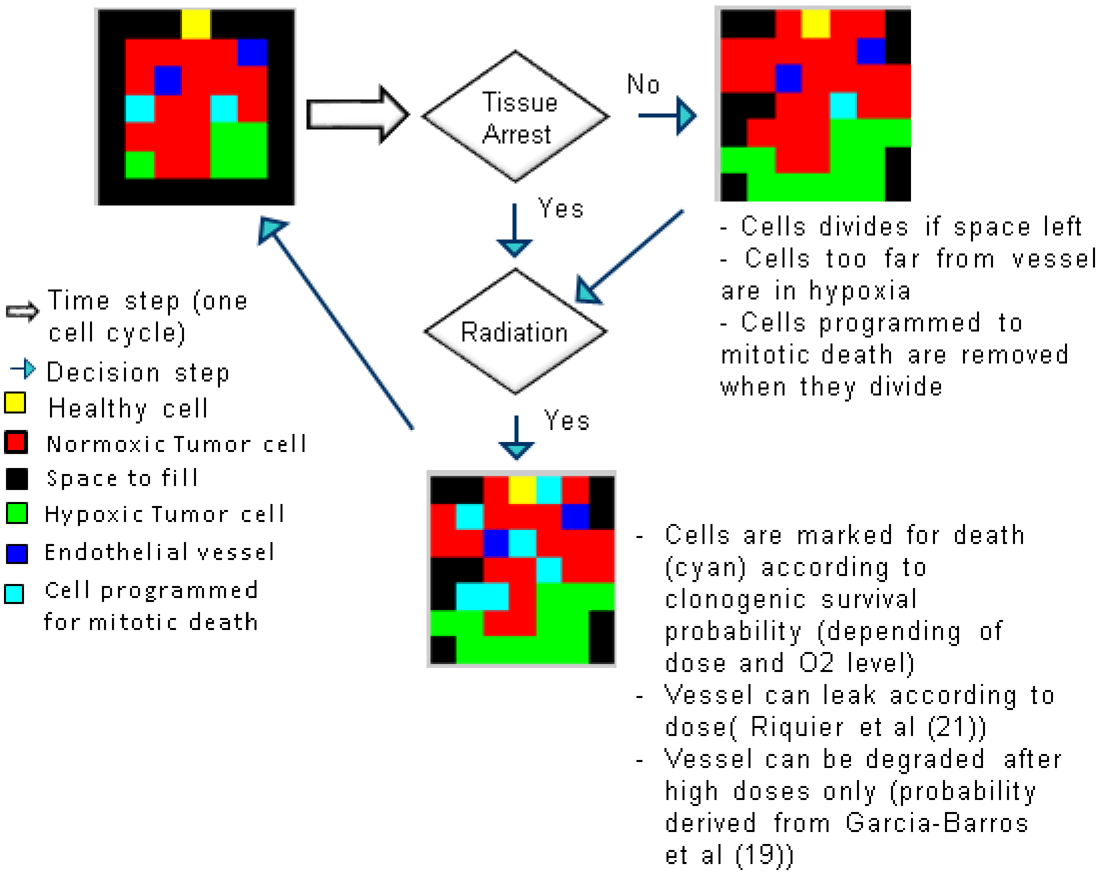

Figure S1 – **Visual flow chart for simulations.**
This flow chart illustrates how cell death and cell division is implemented in the model with respect to time iteration and radiation exposure.

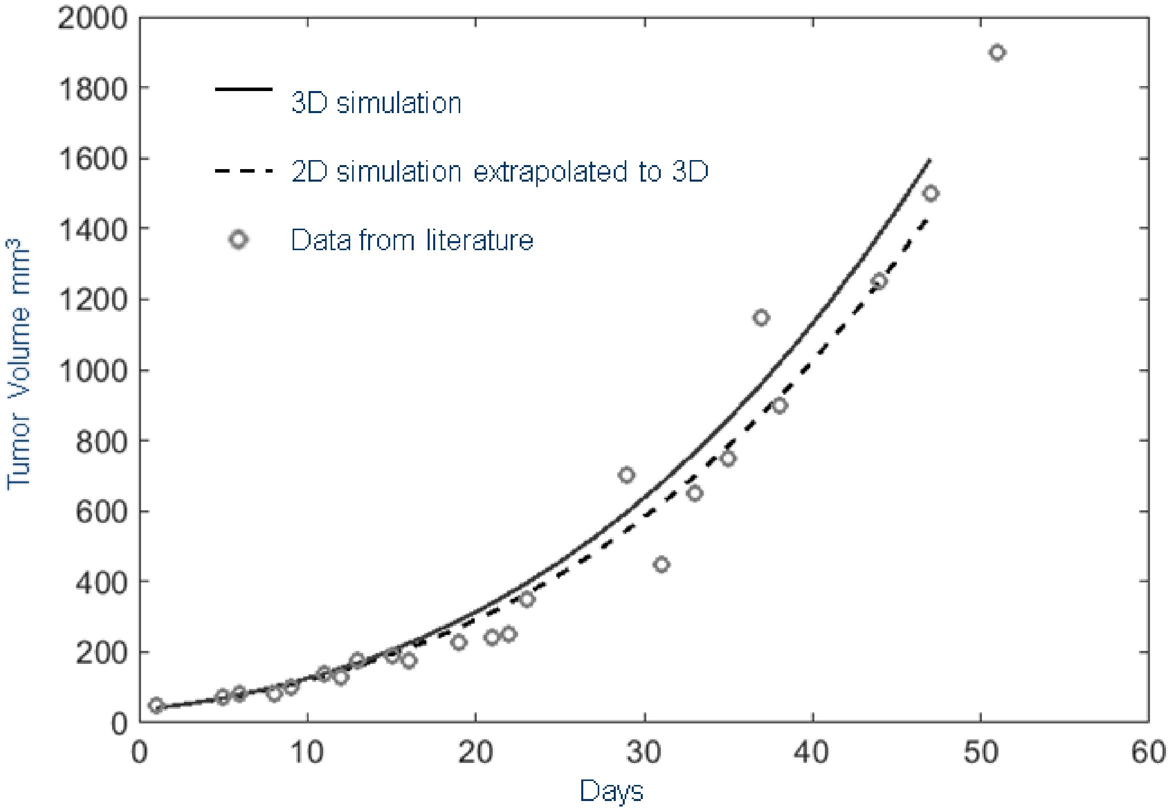

Figure S2 – Comparison of 2D tumour growth corrected to represent volume with 3D tumour growth model

In the 3D tumour growth model, the tumour is represented as a sphere in a 3D isotropic stack over time. We then take advantage of the same algorithm by simply applying image processing in 3D (both dilation and erosion), as explained in the tumour growth model. The results were equivalent to the volume approximation from 2D simulation with the volume estimation from the 2D surface. The 2D simulations are preferred for the sake of simplicity of visualization as well as computing time and memory. Data from the literature are the same as in Fig. 1D[10-12,15].

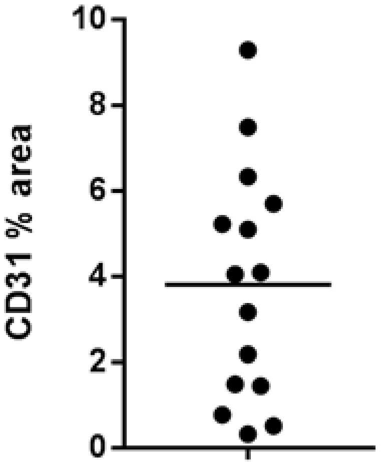

Figure S3: Vessel density in mice tumours

Vessel density was measured on pseudo-confocal images by quantifying the area ratio of tumour-associated blood vessels (CD31+) on 15 tumours from 15 mice, with 2 independent cohorts of mice as non-irradiated controls, as described in [5]. The measured average value of vessel density was 3.8 %.

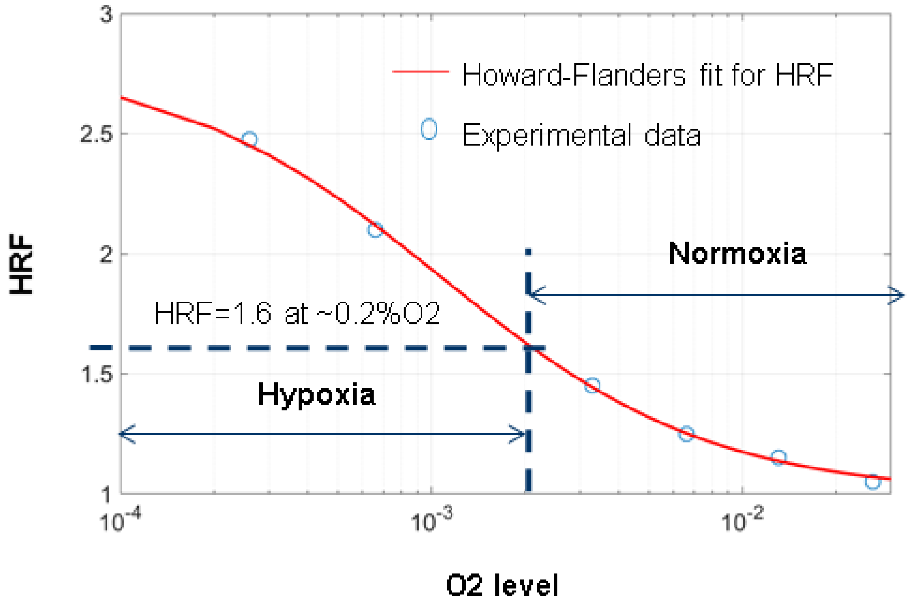

Figure S4: HRF fit.

The Howard-Flanders model is used to fit the HRF, allowing a hypoxia reduction factor (HRF) to be modelled, which was then used to compute the dose equivalent for cell death at each specific oxygenation condition. The dose equivalent for cell death *in vitro* at ambient oxygen, i.e. 21%, was the dose at current oxygen level divided by HRF. The lower the O2 level (hypoxia), the greater the HRF, and therefore the lower the estimated rate of cell death.

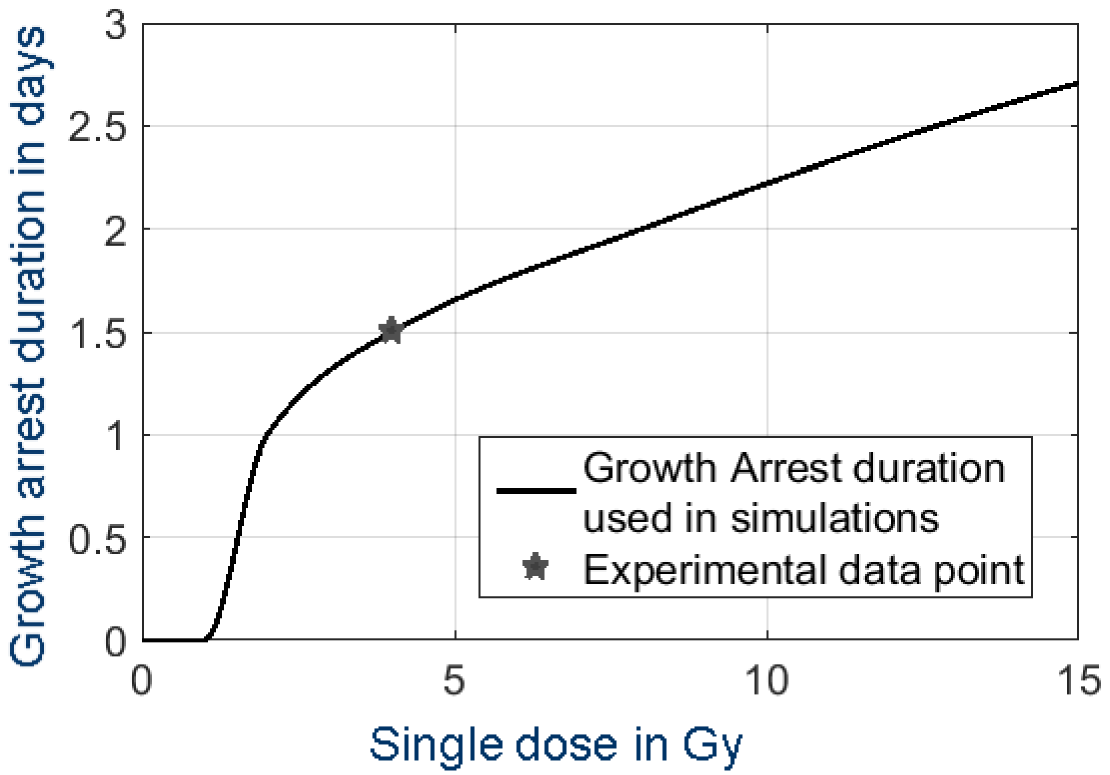

Figure S5: Growth arrest duration used in the simulation.

The values of growth arrest duration (in days) due to single dose irradiation were set to realistic values, with only one measurement reported from a clonogenic assay in [15].

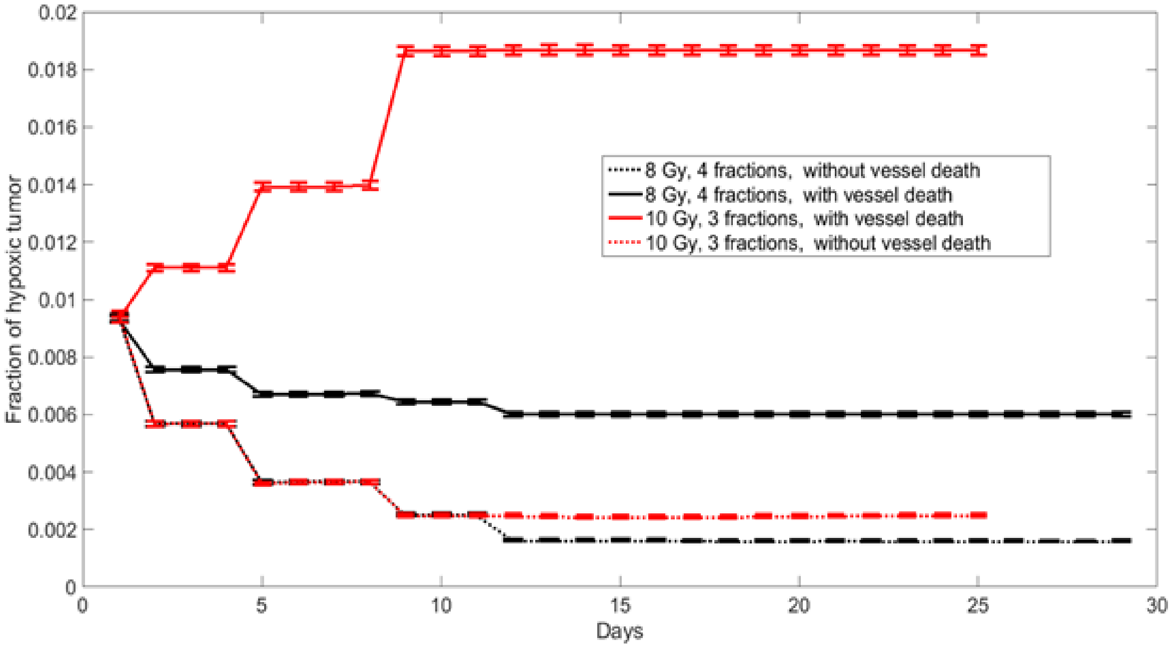

Figure S6: Competition between perfusion and vessel cell death for hypoxic levels in tumour. At high dose (15 Gy), the effect of vessel death as a correction to perfusion hits is more obvious than, say, at 8Gy, and hypoxia dominates the perfusion effect. Note that the error bar does not take into account the fact that most tumours are completely killed at high dose without vessel death, due to less hypoxia, and therefore less tumour resistance.
