# Supplementary material for: Optimizing radiotherapy protocols using computer automata to model tumour cell death as a function of oxygen diffusion processes
Source: Sci Rep. 2017 May 23;7:2280. doi: 10.1038/s41598-017-01757-6 (PMC5442104; doi:10.1038/s41598-017-01757-6)
Supplement: Supplementary file 2 — Supplementary Software [file 41598_2017_1757_MOESM2_ESM.zip › SupSoftware/README_Carot.pdf]

CAROT:

Computational Automata

for Radiotherapy Optimization against Tumor

Companion software for "Optimizing radiotherapy protocols using computer automata to model tumor cell death in function of oxygen diffusion processes."

The model presents the advantage to give a visual feedback and to include for the first time as a working assumption the mitotic death as the principal mode of tumor cell death, and allow to study the effect of hypoxia on different dose, based on experimental data. All the parameters of this model, (listed in supplementary Table 1) are physiologically accessible, and have been fitted to experimental data. A stochastic factor is inserted for the initial spatial disposition of cells, vessel and Oxygen map to model the variability in patient/animal.

The code has been developed under Matlab, and requires the DipImages 2.7

Standalone program:

Some example of output are provided by an exe file for windows 64 bits only, that can be installed by running MyAppInstaller_web.exe, under the condition that an internet access is provided.

Please follow as well the post installation notes given in the Carot Installer (at the end of the installation process).

Some parameters of the simulation can be edited, in particular, the following biological assumptions are taken into account: By checking Hypoxia effect, the diffusion limited oxygenation will be taken into account in the simulation. EC death will also

By checking 'Use a Personalized Protocol', one can test a non-regular scheme of irradiation. A sample text file is provided (exampleprotocol.txt),

8 0 2 2 8

Which mean a 8Gy irradiation on Monday, followed by a one day break, 2 doses of 2 gy, and a last dose of 8 Gy.

Graphical interface:

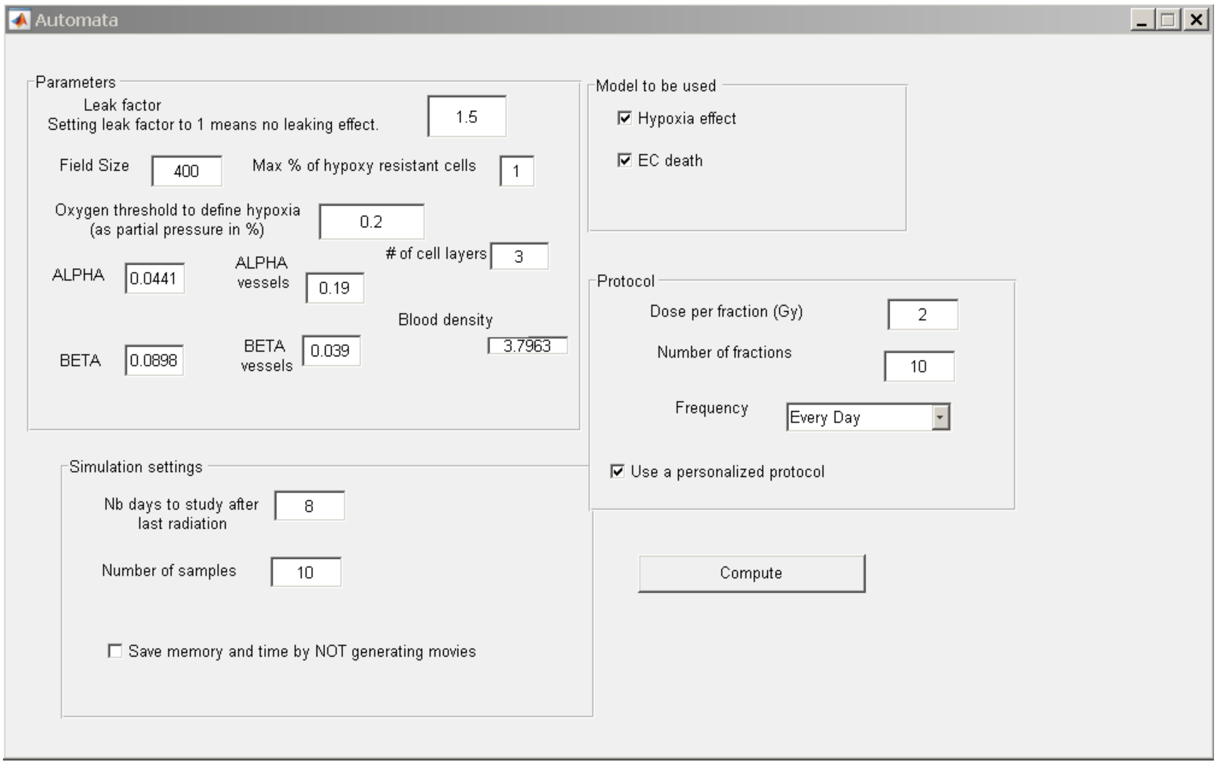

Please be patient in particular if the number of fraction or the number of day after exposure is important.

Simulating 10 samples (10 mice for example) with the personalized protocol described in the example file above , with 8 additional days after the protocol duration, took 59.8 seconds on a double core processor with 16Gb of Ram. A simulation of 10 samples with 10 doses of 2 Gy everyday but on weekends will last 27 seconds.

After having finished the computation, an additional user interface is provided to get some example of outputs.

Example of output: Tumor cell state for one sample, oxygen map, tumor volume over time ith error bar for the 10 samples.

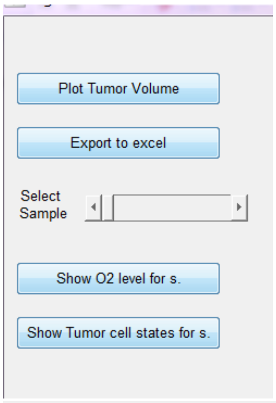

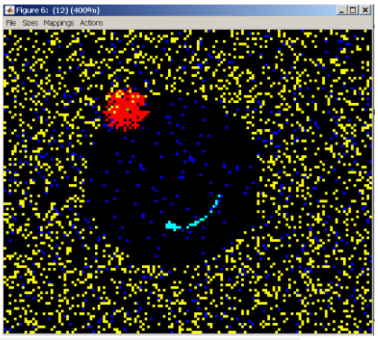

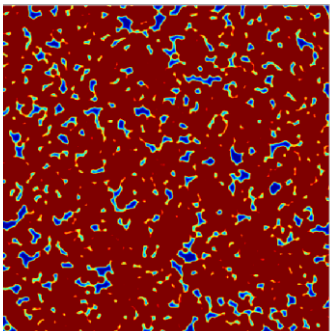

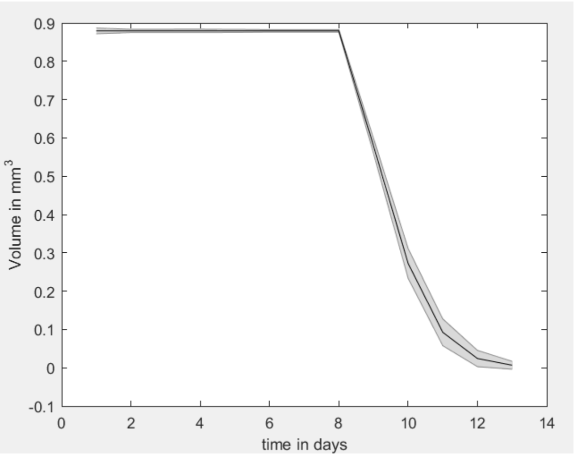

Plot Tumor Volume:

Will plot the average and std of tumor volume (based on a pixel (cell) area of 15um per 15 um)

Export to excel:

Will prompt the user to select a directory where to save

Param.txt (the list of parameters used in this simulation, as filled by the user in the user interface)

Tc.txt : the success of the protocol, meaning the tumor control : if 1 means tuor control, if 0 means no tumor control Can be used to construct Tumor control probabilities curves.

ExportedData.xls: The evolution of cells states over time, averaged over the samples.

Show O2 level for s:

Will display the average level of oxygen in the tumor over time, plus the map of oxygen level over time , and a map of hypoxic area (in red).

To parse the time step, use the ‘n’ (next) and ‘p’ (previous) letters from your keyboard. Some visualization such as zoom facilities or export are provided by Dipimage libs.

Show Tumor cell states for s:

Will display the simulated tumor of sample ‘s’ as selected from the above slide, over time with the following color code:

- red: normoxic tumor cells

- green: hypoxic resistant tumor cells (will divide even in hypoxia)

- dark blue: vessels
- light blue: cell programmed for mitotic death
- yellow: healthy cells.

To parse the time step, use the ‘n’ (next) and ‘p’ (previous) letters from your keyboard. Some visualization such as zoom facilities or export are provided by Dipimage libs.
